# Efficient Production of High-Concentration Poly(3-hydroxybutyrate-*co*-3-hydroxyhexanoate) from CO_2_ Employing the Recombinant of *Cupriavidus necator*

**DOI:** 10.3390/bioengineering12060557

**Published:** 2025-05-22

**Authors:** Kenji Tanaka, Izumi Orita, Toshiaki Fukui

**Affiliations:** 1 Faculty of Humanity-Oriented Science and Engineering, Kindai University, 11-6 Kayanomori, Iizuka-shi 820-8555, Japan; 2 School of Life Science and Technology, Institute of Science Tokyo, 4259 Nagatsuta, Midori-ku, Yokohama 226-8501, Japan; orita.i.bae5@m.isct.ac.jp (I.O.); tfukui@life.isct.ac.jp (T.F.)

**Keywords:** polyhydroxyalkanoate, PHBHHx, CO_2_, *Cupriavidus necator*, hydrogen-oxidizing bacterium, high-cell-density cultivation, gas fermentation

## Abstract

A copolymer of 3-hydroxybutyrate (3HB) and 3-hydoxyhexanoate (3HHx), PHBHHx, is a practical biodegradable plastic, and at present, the copolymer is produced at commercial scale via heterotrophic cultivation of an engineered strain of a facultative hydrogen-oxidizing bacterium, *Cupriavidus necator*, using vegetable oil as the carbon source. In our previous report, we investigated PHBHHx production from CO_2_ via pH-stat jar cultivation of the newly created recombinants of *C. necator* under autotropic conditions, feeding the inorganic substrate gas mixture (H_2_/O_2_/CO_2_ = 80:10:10 *v*/*v*%) into a recycled-gas closed-circuit (RGCC) culture system. The dry cell weight (DCW) and PHBHHx concentration with the best strain MF01/pBPP-ccr_Me_J_Ac_-emd increased to 59.62 ± 3.18 g·L^−1^ and 49.31 ± 3.14 g·L^−1^, respectively, after 216 h. In this study, we investigated the high-concentration production of PHBHHx with a shorter cultivation time by using a jar fermenter equipped with a basket-shaped agitator to enhance oxygen transfer in the culture medium and by continuously supplying the gases with higher O_2_ concentrations to maintain the gas composition within the reservoir at a constant ratio. The concentrations of ammonium and phosphate in the culture medium were maintained at low levels. As a result, the DCW and PHBHHx concentrations increased to 109.5 ± 0.30 g·L^−1^ and 85.2 ± 0.62 g·L^−1^ after 148 h, respectively. The 3HHx composition was 10.1 ± 0.693 mol%, which is suitable for practical applications.

## 1. Introduction

**Figure 1:**
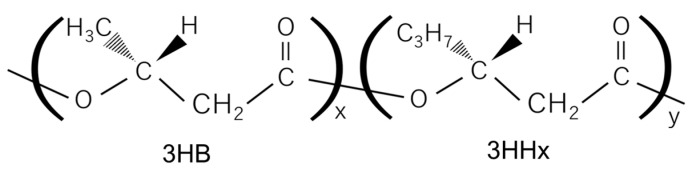
Chemical structure of PHBHHx.

Polyesters of hydroxyalkanoic acids (HAs), polyhydroxyalkanoates (PHAs), synthesized by diverse bacteria, are eco-friendly, biodegradable and thermoplastic polymers. They have the potential to be promising alternatives to petroleum-based polymeric materials. PHAs are superior to many other biodegradable plastics in terms of biodegradability under marine environments [1,2,3]. There is also a review that shows how microbial PHAs, a class of intrinsically natural polymers, can successfully remedy the fossil and persistent plastic dilemma [4]. A homopolyester of (*R*)-3-hydroxybutyric acid, P(3HB), is the firstly discovered and well-studied kind of PHA. Unfortunately, P(3HB) shows stiff and brittle properties [5] and its “processing window” is very narrow, meaning that the melting temperature (T_m_, 170–180 °C) is close to its thermal degradation temperature (180–190 °C) [6]. Copolymers of 3HB and other HAs often exhibit better physical properties than P(3HB). A copolymer of 3HB and (*R*)-3-hydroxyhexanoic acid (3HHx), P(3HB-*co*-3HHx) (PHBHHx), has excellent thermal, mechanical, and physical properties [7]. Figure 1 shows the chemical structure of PHBHHx.

The crystallinity, elasticity, and melting temperature of PHBHHx depends on its 3HHx composition of the copolyester chains. It is known that the copolymer containing ~10 mol% 3HHx shows adequately flexible properties suitable for several practical applications [8]. Many studies have been carried out using a well-known P(3HB) producer, *Cupriavidus necator* (the former names of this bacterium were *Alcaligenes eutrophus*, *Hydrogenomonas eutrophus*, *Ralstonia eutropha*, and *Wautersia eutropha*). Kaneka Corporation (Tokyo, Japan) has developed a commercial bioprocess using a recombinant of *C. necator* to produce PHBHHx from vegetable oil with a capacity of around 5,000 tons/year in 2019 [9] and expanded the production up to 20,000 tons/year [10]. Usually, PHA copolymers are produced in the presence of precursor compounds structurally related to the second monomer unit, such as vegetable oils or fatty acids for the biosynthesis of PHBHHx. A holistic approach has been discussed for these strategies, including the selection of robust microbial strains and feedstock combinations, optimization of cell biomass and biopolymer yields, genetic engineering of biosynthetic pathways, and improvement of downstream processing techniques [11]. Fukui and co-workers have constructed engineered strains of *C. necator* to produce PHBHHx with a high 3HHx composition and a high cellular content from structurally unrelated fructose and glucose, which are inexpensive feedstocks [12,13].

*C. necator* was originally discovered to be a hydrogen-oxidizing bacterium that can also grow chemolithoautotrophically by using H_2_ and O_2_ as the energy source for CO_2_ fixation. Hydrogen-oxidizing bacteria, particularly *C. necator,* are known to be superior in terms of cell growth rate and cell yields on CO_2_ compared to other autotrophic organisms [14]. The interest in *C. necator* and other hydrogen-oxidizing bacteria therefore has emerged for solving global warming problems [15,16,17,18]. Recently, there has been an increase in studies on applications of *C. necator* for converting CO_2_ into useful materials, for instance, sustainable food protein [19], 3-hydroxypropionic acid [20], α-humulene [21], mevalonate [22], resveratrol [23], and l-isoleucine and l-valine [24]. The production of PHAs from CO_2_ by using *C. necator* is highly anticipated as it not only addresses the critical issue of plastic pollution but also offers greater potential for reducing CO_2_ emissions when compared to other materials. We have been studying on the production of P(3HB) and PHBHHx from CO_2_ via autotrophic cultivation of *C. necator* strains and other hydrogen-oxidizing bacteria [25,26,27,28,29,30,31,32,33]. Current reports on cultivation technology for PHA production from CO_2_ by other researchers are also increasing [34,35,36,37,38,39,40,41,42,43,44,45]. It has been frequently demonstrated that *C. nacator* and the related strains produce P(3HB) homopolymer or 3HB-based copolymers containing only faint fractions of other 3HA units from sugars and CO_2_. The studies by Volova et al. also implemented the addition of hexanoate for the synthesis of P(3HB-*co*-13~20 mol% 3HHx) from CO_2_ [46]. This limitation of carbon sources for PHBHHx biosynthesis has been overcome in our previous metabolic engineering research. Our recombinant strains are capable of producing PHBHHx from not only sugars but also CO_2_ as the sole carbon source, and the composition is in the range of 5~50 mol% depending on the modifications and autotrophic conditions, increasing the possibility for larger scale production of the copolymers from CO_2_ [25].

In our previous study [26], we investigated PHBHHx production from CO_2_ in pH-stat jar cultivation of the engineered strains of *C. necator* under autotrophic conditions using a recycled-gas closed-circuit (RGCC) culture system. In the case of recombinant *C. necator* MF01/pBPP-ccr_Me_J_Ac_-emd, the dry cell weight (DCW) and PHBHHx concentration increased to 59.62 ± 3.18 g·L^−1^ and 49.31 ± 3.14 g·L^−1^, respectively, by repeating the exchange of the substrate gas mixture (H_2_/O_2_/CO_2_ = 80:10:10 *v/v*%) within the gas reservoir. The 3HHx fraction in the copolyester was 9.3 mol% in this case, the composition of which was suitable for practical uses as demonstrated previously. However, this cultivation required a long fermentation time (216 h), where the productivity of PHBHHx from CO_2_ was inferior to that obtained by heterotrophic cultivation of other strains engineered by utilizing vegetable oils as carbon sources. Arikawa and Matsumoto [47] reported the construction and evaluation of several gene expression cassettes consisting of promoters and ribosome binding sites that finely regulate the transcription and translation of PHA-related genes. In the jar cultivation of their engineered strains with feeding of palm kernel, the dry cell weight and PHBHHx concentration reached approximately 215 and 175 g·L^−1^, respectively, after 68 h.

In this article, we report the efficient production of PHBHHx from CO_2_ with a higher concentration during a shorter cultivation time compared to our previous study. The strain MF01/pBPP-ccr_Me_J_Ac_-emd was subjected to cultivation using the RGCC culture system and a jar fermenter equipped with a basket-shaped agitator to enhance the gas transfer rate in the culture medium, and the gas mixture with higher O_2_ concentrations was continuously supplied to maintain the gas composition within the gas reservoir at a constant ratio. The procedure for maintaining the concentration of ammonium and phosphate in the culture medium was also optimized. As the result, the yield of cell biomass and the copolymer PHBHHx obtained by these procedures exceeded the known data that were obtained from the autotrophic culture of hydrogen-oxidizing bacteria on a mixture of H_2_/O_2_/CO_2_.

## 2. Materials and Methods

### 2.1. Bacterial Strain

*C. necator* MF01/pBPP-ccr_Me_J_Ac_-emd was used throughout this study. This strain is one of the four recombinants of *C. necator* H16 constructed by Fukui et al. for the biosynthesis of PHBHHx from sugars and CO_2_ [25,26]. Details of the construction of the recombinants were described in our previous reports [12,13,48] (Table 1).

### 2.2. Culture Medium

*C. necator* MF01/pBPP-ccr_Me_J_Ac_-emd was chemolithoautotrophically cultivated in a complete mineral salt medium. The mineral medium for plate culture and flask culture was prepared as follows: 3.0 g (NH_4_)_2_SO_4_ and 0.2 g MgSO_4_·7H_2_O were dissolved in 500 mL tap water, and 4.0 g KH_2_PO_4_, 0.8 g Na_2_HPO_4_, and 1.0 g NaHCO_3_ were dissolved in 489.1 mL distilled water. These two solutions were separately autoclaved at 121 °C for 20 min after their pH was adjusted to 7.0 with 3 M NaOH, respectively. After cooling, they were mixed in a laminar flow cabinet; then, 10 mL of a filter-sterilized 20 mg·mL^−1^ kanamycin solution (final concentration, 200 μg·mL^−1^) and 0.1 mL of a trace-element solution were added. The composition of the trace-element solution was 9.7 g FeCl_3_, 7.8 g CaCl_2_, 0.218 g CoCl_2_·6H_2_O, 0.118 g NiCl_3_·6H_2_O, 0.105 g CrCl_3_·6H_2_O, and 0.156 g CuSO_4_·5H_2_O per 1 L of 0.1 M HCl. This liquid medium was used for flask culture experiments. The agar plate was prepared via the addition of 15 g·L^−1^ agar into the medium and used for subculture as well as refreshment of the stock culture. The mineral salt medium used for jar cultivation was prepared by altering the concentration of the phosphate salts and (NH_4_)_2_SO_4_.

### 2.3. Revival of Recombinant Strain and Preculture

The cells from a glycerol stock of the strain MF01/pBPP-ccr_Me_J_Ac_-emd stored at −80 °C were revived on the mineral agar plate. They were inoculated on the surface of the agar plate with an inoculating loop and then put inside a vacuum desiccator (volume size 6 L). The air was evacuated from the desiccator by using a vacuum pump; then, the substrate gases were introduced from the cylinders at a ratio of H_2_/O_2_/CO_2_ = 80:10:10 *v*/*v*% by reading the indication of a vacuum gage. The desiccator was incubated at 30 °C for 72 h for cell growth.

The seed for jar cultivation was prepared via flask culture under autotrophic conditions. One loop of the cells grown on the agar plate was inoculated with 15 mL of the liquid mineral medium in a 300 mL Erlenmeyer flask. The flask was plugged with a silicon rubber stopper which was penetrated with a glass tube connected to a sterile filter (pore size, 0.2 μm) and silicone rubber tube. The air within the flask was evacuated through the sterile filter and then the gases were introduced as described above. The flask was reciprocally shaken at a speed of 170 rpm and 30 °C for 72 h.

### 2.4. Culture System

**Figure 2:**
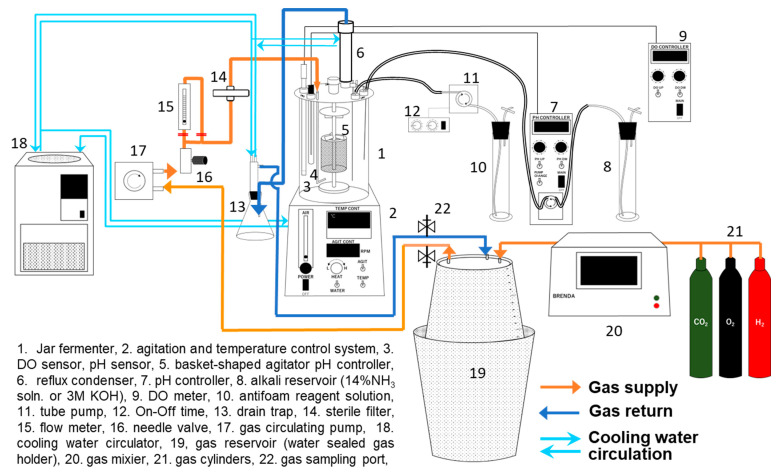
The RGCC culture system for high-cell-density cultivation of *C. necator* MF01/pBPP-ccr_Me_J_Ac_-emd under chemolithoautotrophic conditions.

High-cell-density cultivation of MF01/pBPP-ccr_Me_J_Ac_-emd under autotrophic conditions was carried out using a 1 L scale jar fermenter (BMJ, Biott Co., Ltd., Tokyo, Japan) and a recycled-gas closed-circuit (RGCC) culture system (Figure 2). The RGCC system was developed for the chemoautotrophic cultivation of hydrogen-oxidizing bacteria, allowing us to reduce the loss in substrate gas usage [49,50,51]. We used the RGCC system for the study on PHA production from CO_2_ with *C. necator* and other hydrogen-oxidizing bacteria [26,27,28,29,30,31,32,33].

In this study, a basket-shaped agitation unit (EG-STAR, Biott Co., Ltd.) was used within the 1 L-scale jar fermenter to enhance the mass transfer of the substrate gases in the mineral medium. The volume coefficient of the mass transfer for oxygen (*k*_L_*a*) measured by using the sulfite oxidation method was 450 h^−1^ at 650 rpm and a feed rate of 0.6 vvm.

A handmade water-sealed gas holder with a large headspace capacity was used as the reservoir for the substrate gas mixture. The detailed descriptions can be found in our previous report [26]. It was assembled with a plastic bucket (total volume of 60 L) that was floated upside down in a larger plastic bucket (total volume of 100 L) containing saturated salt water. The volumetric capacity of the two buckets was enlarged compared to that of a previous study. The gases were introduced from the cylinders into the smaller bucket after the inside air was evacuated. In order to achieve the desired gas composition, the proper volume of each substrate gas was introduced into the holder by reading the depth of the smaller bucket sinking in the saline water holder (the larger bucket) via a ruler marked on the surface. The gas mixture within the reservoir was fed into the medium within the fermenter near the inlet by using a diaphragm pump. The unconsumed gas exhausted from the fermenter was recycled by using a sterile filter in the reservoir. The gas mixture was passed through sterile filter units (pore size 0.2 μm) which were installed at the gas tracts near the inlet and outlet of the fermenter for recycling within the RGCC system. Although strict sterility in the system was not ensured, contamination seems to be unlikely under obligative autotrophic conditions. Indeed, we have not experienced such troubles even during long-term cultivation.

The gas composition of H_2_/O_2_/CO_2_ = 80:10:10 *v/v*% was usually set at the start of cultivation considering that most hydrogen-oxidizing bacteria including *C. necator* are sensitive to oxygen and their optimal O_2_ concentration is much lower than that in the atmosphere [18]. The concentration of O_2_ and CO_2_ in the gas mixture within the reservoir drastically decreased during the cultivation, while that of H_2_ increased a little, because the ratios of O_2_ and CO_2_ to H_2_ in the gas mixture of H_2_/O_2_/CO_2_ = 80:10:10 *v/v*% are smaller than those of the gas consumed according to the stoichiometry described above [26]. The oxygen transfer rate in the culture medium decreased in association with the decrease in the O_2_ composition of the gas mixture, which diminished the production rate of the cells and PHA. Hence, the headspace gas in the reservoir was eliminated by evacuation and refilled with fresh gases before the O_2_ concentration decreased to 5 *v/v*% in order to reset the gas composition. Further, a gas mixer equipped with mass flow controllers (BRENDA BR-3C, KOFLOC Corp., Kyoto, Japan) was used to continuously supply the gas mixture into the reservoir with a fixed composition at a constant flow rate, with the exchange of the reservoir gas frequently required due to the high gas consumption rate by the high-density cells. The composition of the gas mixture within the fermentation system was analyzed several times per day by gas chromatography, as described in a later paragraph, according to the change in residual volume of the gas mixture within the gas reservoir (water-sealed gas holder floating in saline water). The analysis of gas composition was carried out immediately before and after the gas mixture within the reservoir was exchanged for supplementing the gases and/or resetting the composition. When exchanging the gas mixture, the residual gas within the reservoir was evacuated, and then H_2_, O_2_, and CO_2_ were supplied in that order from the cylinders until the corresponding volumes to the targeted gas composition were achieved.

### 2.5. Conditions for Jar Cultivation

The working volume of the mineral salt medium at the start of jar cultivation was set to 600 mL. The cultivation was started by feeding the substrate gas mixture at a feed rate of 360 mL·min^−1^ (equivalent to 0.6 vvm) after inoculating 15 mL of the culture broth prepared via flask culture, as described in the previous paragraph. The agitation speed was set to 300 rpm at the start of cultivation, and then it was stepwisely raised up to 650 rpm while monitoring the decrease in the dissolved oxygen concentration. The pH was maintained at 7.0 by automatically adding 3 M KOH or a 14 *w*/*w*% aqueous solution of ammonium hydroxide (NH_4_OH) with a pH controller (PHC-2201, Biott Co., Ltd.). The NH_4_OH solution was added not only to maintain the culture pH but also to supplement ammonium as the nitrogen source. In high-cell-density cultivations, a small amount of 0.1 g·mL^−1^ KH_2_PO_4_ solution, 0.1 g·mL^−1^ MgSO_4_·7H_2_O solution, and a 10-fold diluted trace-element solution were added for the supplementation of the phosphorus source, Mg^2+^, and microelements like Fe^2+^, respectively, according to the increase in cell concentration. To prevent the culture broth from flowing out of the fermenter due to foaming, a few drops of a diluted antifoaming reagent (type-L, FUJIFILM Wako Pure Chemical Corporation, Osaka, Japan) were repeatedly added to the culture broth during the cultivation.

### 2.6. Analyses

The cell concentration was determined by measuring the optical density at a wavelength of 600 nm (OD_600_) of the culture broth sampled from the fermenter. The dissolved oxygen concentration (DO) of the culture medium was monitored with a DO sensor (SDOU, Biott Co., Ltd.) and a DO meter (DJ-1033, Biott Co., Ltd.). Although it is known that the presence of hydrogen does not allow for the correct measurement of the oxygen concentration, we confirmed that the SDOU-type DO sensor responds to some degree to the change in O_2_ concentration under H_2_-rich conditions. In this study, the DO sensor was used to roughly check if the DO was depleted under autotrophic conditions. The concentration of NH_4_^+^ in the supernatant of the culture broth was determined using the indophenol blue colorimetric method and that of PO_4_^3-^ was determined using the molybdenum blue colorimetric method.

The gas mixture was sampled from the gas reservoir, the inlet of the fermenter after passing through the pump, and the outlet. We then used a gas chromatograph (GC-2014, Shimadzu Corporation, Kyoto, Japan) equipped with a thermal conductivity detector and a 4 mm × 6 m column, into which 5A molecular sieves and Porapack Q were packed, for determination of the gas composition. The composition of the gas mixture taken from the inlet was almost the same as that of the gas reservoir.

For determination of the dry cell weight (DCW), an aliquot of the culture broth was heated in boiling water for the deactivation of PHA depolymerase; then, it was centrifuged and washed with distilled water. The collected cells were dried at 105 °C for 24 h and then the DCW was measured. The content of PHBHHx and the monomer composition of the polymer in the dry cells were determined by using a gas chromatograph (GC-2014) equipped with a InertCap-1 capillary column (30 m × 0.25 mm) and a flame ionization detector. The culture broth, after the heat treatment in boiling water, was centrifuged, and the harvested cells were subjected to lyophilization at −55 °C. The lyophilized cells were suspended in a methanol and chloroform solution containing 15% sulfuric acid, and then treated with direct methanolysis at 100 °C for 140 min. The amount of methyl esters of 3HB and 3HHx formed in the acidic methanol solution were determined by gas chromatography, as described previously [52]. All the measurements were performed in triplicate. In the jar cultivation of *C. necator* under autotrophic conditions, the culture broth within the fermenter was gradually diluted by the accumulation of the pH neutralizer and the water generated along with hydrogen oxidation. The data for the culture broth and its supernatant shown in this report are not compensated in terms of dilution within the fermenter.

## 3. Results

### 3.1. Jar Cultivation Using Synthetic Media with Different Compositions

Cell growth and polymer accumulation of hydrogen-oxidizing bacteria depends on the composition of the mineral medium, particularly the concentration of nitrogen (ammonium) and phosphorus (phosphate). It is known that high concentrations of ammonium inhibit the cell growth of *C. necator,* while low concentrations of ammonium allow for high contents of the polymer in the cells. It is difficult to obtain a high cell concentration when using media with low concentrations of ammonium and phosphate. Therefore, the cultivation tests under pH-stabilized autotrophic conditions were carried out using mineral media with a wide range of concentrations of ammonium and phosphate. All of the cultivations were started by feeding the gas mixture with a composition of H_2_/O_2_/CO_2_ = 80:10:10 *v*/*v*%. The pH within the fermenter was automatically maintained at 7.0 by adding 3 M KOH during the cultivation. The concentrations of (NH_4_)_2_SO_4_ and KH_2_PO_4_ in the medium at the start of cultivation were altered as shown in Table 2. The concentrations of other components were fixed as follows: MgSO_4_·7H_2_O·0.2 g·L^−1^, NaHCO_3_ 0.5 g·L^−1^ and the trace-element solution 0.1 mL·L^−1^. The results are summarized in Table 2.

The cell concentration (OD_600_) of MF01/pBPP-ccr_Me_J_Ac_-emd tended to increase as (NH_4_)_2_SO_4_ concentration increased, except for the cultivation with 10 g·L^−1^ (NH_4_)_2_SO_4_. The highest cell concentration among all of the cultivations examined was obtained with 5.0 g·L^−1^ (NH_4_)_2_SO_4_ and 0.5 g·L^−1^ KH_2_PO_4_. The cell concentration and the cellular PHBHHx content in the cultivations with 10 g·L^−1^ (NH_4_)_2_SO_4_ were lower than those with 5.0 g·L^−1^ (NH_4_)_2_SO_4_. It is known that ammonium has strong toxic and mutagenic effects on microbial cells. The effect on the ammonium concentration in the culture of *C. necator* is extreme in contrast to potassium, phosphorus, magnesium, potassium, sodium, and iron, which have wide saturation zones. It is also known that NaOH and KOH accumulated in the culture medium are toxic for bacterial cells. The amount of KOH solution added in the cultivation with 10 g·L^−1^ (NH_4_)_2_SO_4_ and 0.5 g·L^−1^ KH_2_PO_4_ was 36 mL, while that with 5 g·L^−1^ (NH_4_)_2_SO_4_ and 0.5 g·L^−1^ KH_2_PO_4_ was 18 mL. It seems that in the cultivation with 10 g·L^−1^ (NH_4_)_2_SO_4_, the cell growth and/or polymer accumulation were suppressed by the high concentrations of ammonium and larger amount of the added 3 M KOH.

PHA is usually synthesized as stored carbon and energy under conditions of nutrient limitation, particularly limited N-source, P-source, or DO in the culture medium. When PHA accumulation is suppressed, a sufficient amount of nutrients are available. It should be noted that adequate supplementation of N- and P-sources along with other mineral nutrients in the medium is essential to enable cell growth as well as to support cell viability by maintaining physiological functions such as the activation of enzymes in order to achieve high-cell-density cultivation. In the heterotrophic culture with an organic carbon (C) source, a very high cell concentration with a high content of PHA is often achieved by feeding the C-source solution with a high C/N ratio [53] or a high C/P ratio [54]. In this study, we were not able to determine the appropriate values of C/N and C/P ratios for the efficient production of cells and PHBHHx under autotrophic conditions. Also, the procedure for feeding CO_2_ and the other gases to maintain the C/N or C/P ratios was not sufficient. This will be achieved by more precisely monitoring the rates of gas consumption during cultivation.

### 3.2. Jar Cultivation with 3 M KOH, 14 w/w% NH_4_OH, and Phosphate

**Figure 3:**
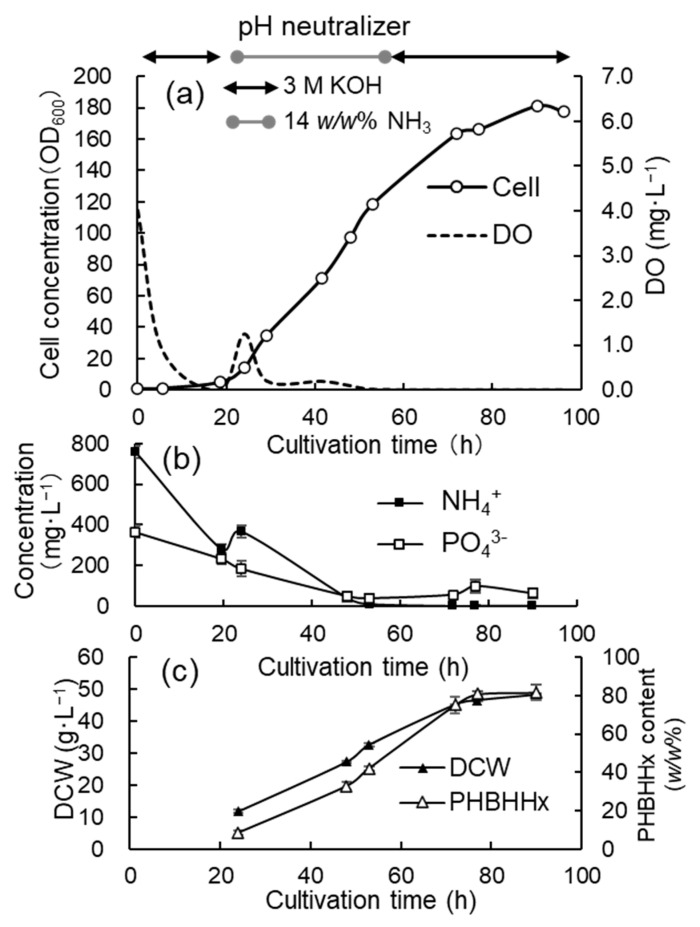
Time courses for the high-cell-density cultivation of *C. necator* MF01/pBPP-ccr_Me_J_Ac_-emd under autotrophic conditions adding 3 M KOH, 14 *w/w*% NH_4_OH, and phosphate. The changes in the cell concentration (OD_600_) and DO (**a**), the concentrations of NH_4_^+^ and PO_4_^3−^ (**b**), and the DCW and cellular content of PHBHHx (**c**).

To increase the cell concentrations and cellular content of PHBHHx, 3 M KOH and a 14 *w*/*w*% NH_4_OH were alternately added to the culture medium to maintain the NH_4_^+^ concentration at low levels. From the start to 18.5 h, the KOH solution was added and then the NH_4_OH solution was added from 18.5 h to 54 h. After that, 3 M KOH was used again until the end of cultivation. The volume of 3 M KOH added was 12.5 mL and that of the ammonium hydroxide solution was 23 mL during the cultivation. As the cell concentration shown by OD_600_ increased by about 30, a phosphate solution (0.1 g·mL^−1^ KH_2_PO_4_) was repeatedly added to the culture medium at a concentration equivalent to 0.1 g·L^−1^ through a sterile filter. The addition of the phosphate solution was implemented 6 times at 28 h, 42.5 h, 51 h, 55 h, 68 h, and 79 h. Figure 3a shows the time course for the cell concentration (OD_600_) of the culture broth and DO values. The specific growth rate was 0.10–0.13 h^−1^ from 6 h to 29 h, and then it gradually decreased as the cell concentration increased. In the late stage of the cultivation (72–96 h), the growth rate was 0.07–0.06 h^−1^.

Figure 3b,c show the time courses for the concentrations of NH_4_^+^ and PO_4_^3−^, as well as the DCW and cellular content of PHBHHx, respectively. In this cultivation, DO decreased to an undetectably low level after 20 h of cultivation, and then the gas mixture within the reservoir was exchanged to increase the O_2_ concentration to 15 *v/v*%. OD_600_ increased to 180.9 after 90 h of cultivation with DCW at 48.24 g·L^−1^. The concentration of NH_4_^+^ decreased to an undetectable level after the addition of the NH_4_OH solution was stopped. The concentration of PO_4_^3-^ decreased to 48.3 mg·L^−1^ after 48 h and then it was maintained within the level from 40 mg·L^−1^ to 100 mg·L^−1^ via the addition of the phosphate solution. The cellular content of PHBHHx increased after 24 h, which was promoted by the DO limitation and the shortage of NH_4_^+^ in the medium. The cellular content of PHBHHx at the end of cultivation reached up to 81.8 ± 3.4 *w/w*% and the 3HHx composition was 9.6 ± 1.1 *w/w*%. It was suspected that the increase in the cell concentration and PHBHHx content ceased due to the excessive addition of phosphate.

### 3.3. Jar Cultivation with Limited Addition of Phosphate

**Figure 4:**
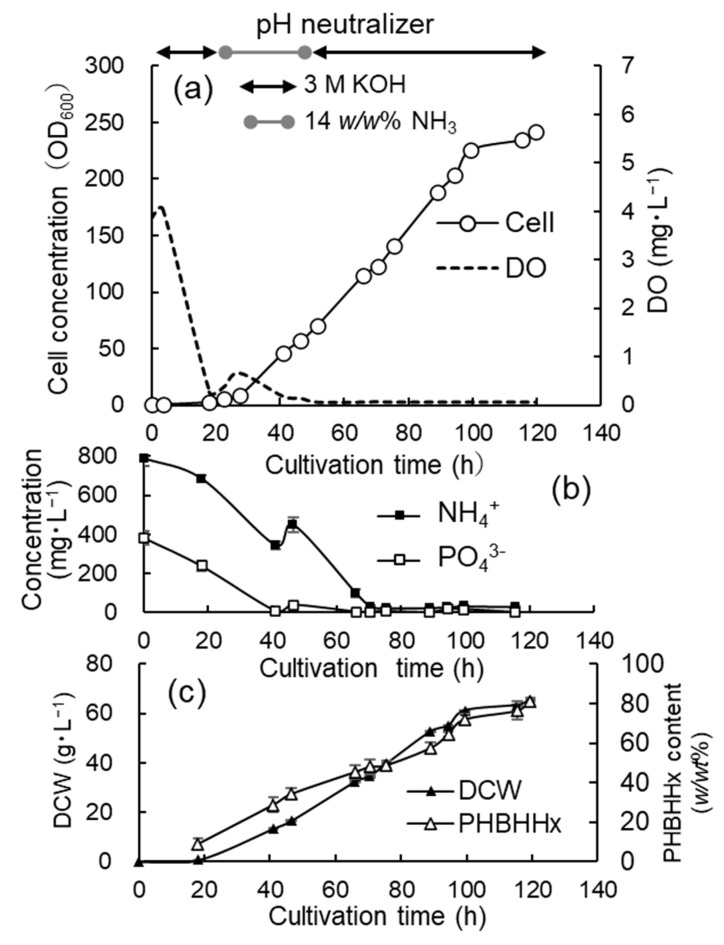
Time courses for the high-cell-density cultivation of *C. necator* MF01/pBPP-ccr_Me_J_Ac_-emd under autotrophic conditions with limited addition of phosphate. The pH neutralizers were 3 M KOH and 14 *w/w*% NH_4_OH. The changes in the cell concentration (OD_600_) and DO (**a**), the concentrations of NH_4_^+^ and PO_4_^3−^ (**b**), and the DCW and cellular content of PHBHHx (**c**).

The next cultivation was carried out with the addition of a reduced amount of phosphate. As OD_600_ increased by about 60, the phosphate solution (0.1 g·mL^−1^ KH_2_PO_4_) was repeatedly added to the culture medium in an amount equivalent to 0.1 g·L^−1^. The addition of the phosphate solution was implemented 4 times at 48.5 h, 67.5 h, 90 h, and 116 h. The pH neutralizer used was 3 M KOH, incorporated from the start to 20 h and from 53.5 h until the end of cultivation, and 14 *w/w*% NH_4_OH was used from 20 h to 53.5 h. The volume of the KOH solution added was 21 mL and that of the NH_4_OH solution was 19.5 mL. The time courses for OD_600_ and DO, the concentrations of NH_4_^+^ and PO_4_^3−^, and the DCW and cellular content of PHBHHx are shown in Figure 4a, Figure 4b, and Figure 4c, respectively. The specific growth rate was 0.10–0.12 h^−1^ from 4 h to 41 h, and then it gradually decreased as the cell concentration increased. In the late stage of cultivation (89–120 h), the growth rate was 0.07–0.05 h^−1^.

OD_600_ reached 242 at the end of cultivation, and the DCW increased up to 65.2 ± 0.16 g·L^−1^. The cellular content of PHBHHx was 81.3 ± 1.9 *w/w*% and the 3HHx composition was 12.5 ± 0.4 *w/w*%. The exchange of the gas mixture within the reservoir was frequently repeated after 95 h; however, the O_2_ concentration sharply decreased within a few hours. Consequently, the production of PHBHHx was suppressed.

### 3.4. Jar Cultivation with Continuous Supply of O_2_-Rich Gas Mixture to Gas Reservoir

**Figure 5:**
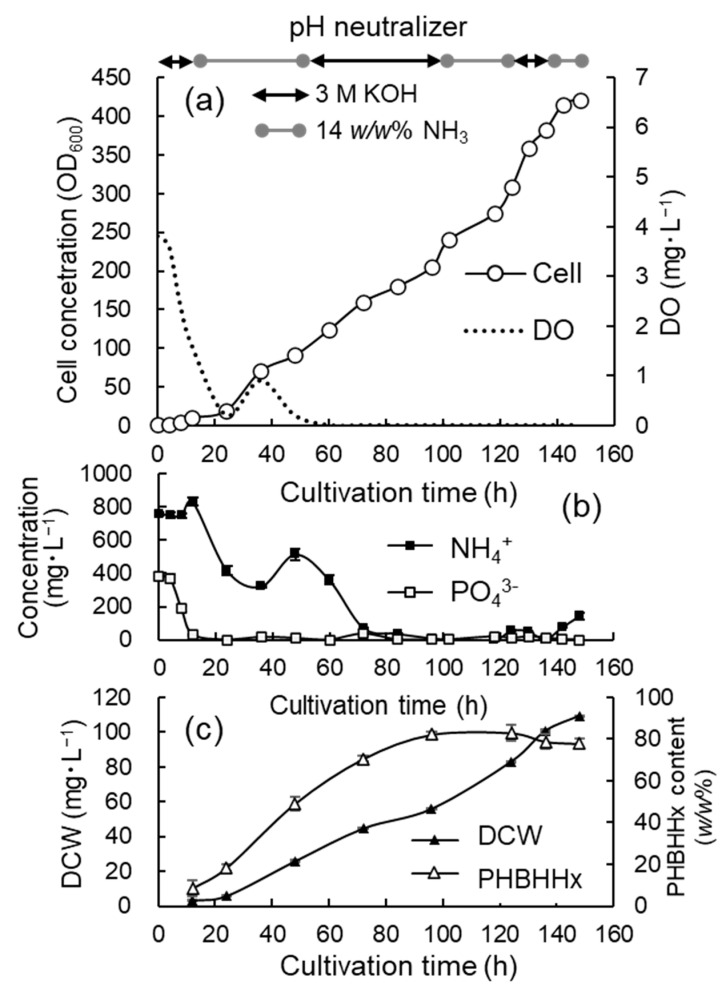
Time courses for the high-cell-density cultivation of *C. necator* MF01/pBPP-ccr_Me_J_Ac_-emd under autotrophic conditions with continuous supplying of an O_2_-rich gas mixture to the gas reservoir. The pH neutralizers were 3 M KOH and 14 *w/w*% NH_4_OH. The changes in the cell concentration (OD_600_) and DO (**a**), the concentrations of NH_4_^+^ and PO_4_^3−^ (**b**), and the DCW and cellular content of PHBHHx (**c**).

**Figure 6:**
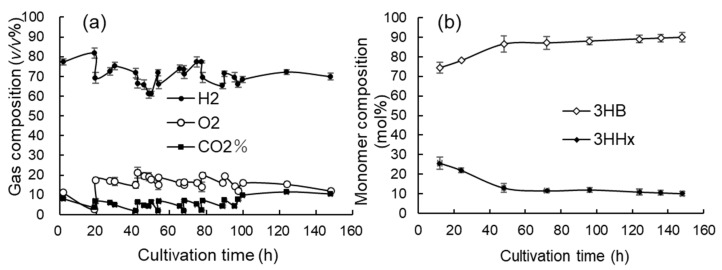
Time courses for the high-cell-density cultivation of *C. necator* MF01/pBPP-ccr_Me_J_Ac_-emd under autotrophic conditions with continuous supplying of an O_2_-rich gas mixture to the gas reservoir. The pH neutralizers were 3 M KOH and 14 *w/w*% NH_4_OH. The changes in gas composition within the gas reservoir (**a**); the monomer composition of PHBHHx accumulated in the cells (**b**).

To prevent the shortage of the substrate gases, particularly oxygen, within the reservoir, a gas mixture with a fixed composition was continuously supplied to the reservoir through a gas mixer starting from the mid-term of cultivation. The results are shown in Figure 5a–c and Figure 6a,b.

The gas composition within the reservoir was set to H_2_/O_2_/CO_2_ = 80:10:10 *v*/*v*% at the start of the cultivation. At 19.5 h, 30 h, 41 h, 54 h, and 68 h, the gas in the reservoir headspace was batchwise refreshed with the gas mixture of H_2_/O_2_/CO_2_ = 72:18:10 *v*/*v*%. From 68 h to 100 h, the gas mixture with a composition of H_2_/O_2_/CO_2_ = 70:20:10 *v*/*v*% was continuously supplied through the gas mixer to the reservoir at a flow rate of 70 mL·min^−1^. The exchange of headspace gas to reset the composition to 70:20:10 *v*/*v*% was again carried out at 78 h and 90 h. From 100 h until the end, the gas mixture with a composition of H_2_/O_2_/CO_2_ = 68:20:12 *v*/*v*% was continuously supplied at a flow rate of 90 mL·min^−1^ without exchanging the gas mixture. The pH neutralizer used was 14 *w*/*w*% NH_4_OH from 4 h to 60 h, from 102 h to 124 h, and from 136 h to 148 h, and 3 M KOH from 0 h to 4 h, from 60 h to 102 h, and from 124 h to 136 h. The volume of the KOH solution added during the cultivation was 9 mL and that of the NH_4_OH solution was 33 mL. The phosphate solution was added at 37 h, 61 h, 97 h, 118 h, and 130 h. A magnesium sulfate solution (0.1 g·mL^−1^ MgSO_4_·7H_2_O) was added at 49 h, 85 h, and 118 h in an amount equivalent to 0.6 g·L^−1^. Further, a 10-fold diluted trace-element solution was added twice for the supplementation of microelements such as Fe^2+^ in an amount equivalent to 0.5 mL·L^−1^ at 72 h and 118 h. To achieve a higher initial cell concentration at the start of cultivation, the inoculation of the seed culture was increased to 30 mL. After 148 h, OD_600_ reached a very high value of 421. The specific growth rate was 0.17–0.12 h^−1^ from 8 h to 24 h, and then it gradually decreased as the cell concentration increased. In the late stage of cultivation (102 h–148 h), the growth rate was 0.05–0.04 h^−1^. The DCW and PHBHHx concentration increased up to 109.5 ± 0.30 g·L^−1^ and 85.2 ± 0.62 g·L^−1^, respectively, and the cellular content of PHBHHx was 77.8 ± 2.5 *w/w*% (Figure 5a,c). The productivities of the cells and PHBHHx were 0.740 ± 0.002 g·L^−1^·h^−1^ and 0.576 ± 0.004 g·L^−1^·h^−1^, respectively. The concentration of NH_4_^+^ was maintained at very low levels from 70 h to 120 h, and the concentration of PO_4_^3−^ was maintained at less than 20 mg·L^−1^ until the end of the cultivation (Figure 5b).

The time course for the gas composition in the reservoir is shown in Figure 6a. After 100 h, the composition of the substrate gas mixture was maintained at a roughly constant level. The altered composition of the gas mixture after 100 h (H_2_/O_2_/CO_2_ = 68:20:12 *v*/*v*%) was designed by making slight adjustments to the ratio of the gas species (H_2_/O_2_/O_2_ = 67.35: 24.49: 8.16 *v/v*%) based on the stoichiometry for P(3HB) production (2) described in a later paragraph. It was thought that the constant ratio of the gas mixture indicated that the supply of the gas species was well balanced with cellular consumption within the fermenter. The time courses for the 3HHx composition of the polymer accumulated in the cells are shown in Figure 6b. In the early stage of the culture, the 3HHx composition was markedly high at 25.6 ± 3.1 mol% at 12 h, and then gradually decreased as the cell concentration increased and remained almost constant at around 10 mol% after 70 h. The 3HHx composition at the end of the cultivation was 10.1 ± 0.693 mol%.

## 4. Discussion

In this study, the ratio of the substrate gases within the gas reservoir was almost constantly maintained after the mid-term of cultivation by continuously supplying the gas mixture with the appropriate composition, which enabled us to achieve a very high cell concentration without the frequent exchanging of the gas mixture within the reservoir (Figure 5 and Figure 6). The supplementation of the substrate gases and the control of the gas composition in gas-recycling cultivation systems in our procedure will be essential for reducing the size of gas reservoirs in the commercial production of biodegradable polymers from CO_2_ employing hydrogen-oxidizing bacteria.

**Figure 7:**
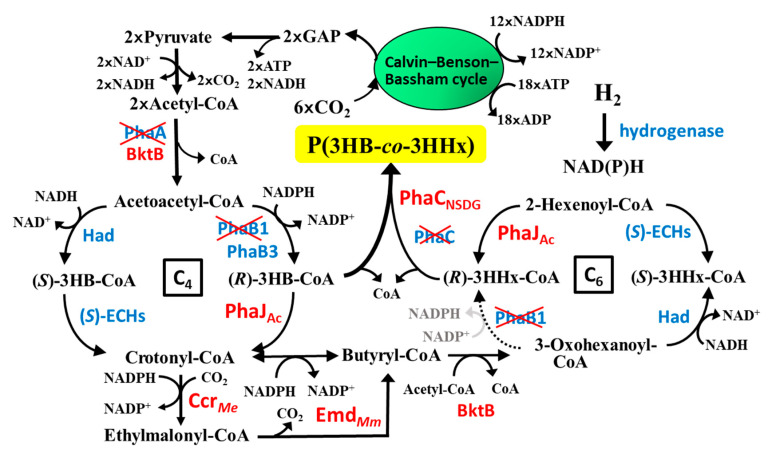
A proposed pathway for PHBHHx biosynthesis from CO_2_ in *C. necator* MF01ΔB1/pBPP-ccr_Me_J_Ac_-emd.

The results of this study demonstrated that the 3HHx unit was efficiently synthesized and incorporated into the copolymer in the strain MF01ΔB1/pBPP-ccr_Me_J_Ac_-emd. A proposed pathway for PHBHHx biosynthesis from CO_2_ in the strain is shown in Figure 7. Recently, the biosynthesis of the copolymer PHA from CO_2_ as the sole carbon source has been reported by some researchers. Fukui et al. succeeded in increasing the 3HHx fraction in P(3HB-*co*-3HHx) from 12.5 mol% up to 19.6 mol% in gas fermentation combined with water electrolysis of recombinant *C. necator* by overexpression of cytosolic carbonic anhydrase [55]. It was also reported that recombinant *R. eutropha* 1F2 harboring *phaCAc_NSDG* synthesized 3HB-based PHA copolymers containing 3-hydroxyvalerate (3HV) and 3-hydroxy-4-methyvalerate (3H4MV) comonomer units under obligative autotrophic conditions by supplying a low-hydrogen-content gas mixture (3.8% H_2_, 7.3% O_2_, 13.0% CO_2_, and 75.9% N_2_), and several additional modifications enhanced the 3HV fraction up to 6.4 mol% using CO_2_ [56].

The reason for the change in the 3HHx composition during the cultivation (Figure 6b) is not experimentally elucidated but it can be deduced as follows. There may be some drastic changes in the PHBH biosynthesis system between the exponential growth phase and PHA accumulation phase not associated with cell division, which is caused by a shortage of ammonium and/or phosphate. It is known that in the polymer synthesis mode, the PHA cycle functions in cells, including a set of enzymes of synthesis and endogenous degradation. As the culture ages and approaches the stationary phase, the activity of depolymerization enzymes increases, and the C-chain of the C_6_ monomers cleaves into C_4_ and C_2_ fragments. The C_2_ fragments, acetyl-CoAs, are resynthesized into (*R*)-3HB-CoA, which increase in the copolymer contrary to a decrease in the (*R*)-3HHx monomers.

In the autotrophic cultivation of *C. necator*, NH_4_^+^ is the most consumed compound among the components of the mineral salt medium. When (NH_4_)_2_SO_4_ was used as the nitrogen source, SO_4_^2−^ remained in the medium after exhaustion of NH_4_^+^ led to a decrease in the pH of the culture medium. The use of 14 *v/v*% NH_4_OH as the pH neutralizer allowed not only for the adjustment of the pH of the medium but also for the supply of an additional N-source to support the further growth of the cells. However, excessive addition of NH_4_OH often inhibits PHA accumulation, especially under DO-limited conditions. Although the addition of NH_4_OH should be carefully controlled to avoid depletion while maintaining a low concentration of NH_4_^+^ in the culture medium, the pH of the medium is influenced by various factors, including not only the balance between NH_4_^+^ and SO_4_^2−^ but also other inorganic ions and the dissolved CO_2_ concentrations. In addition, the cellular consumption of the inorganic compounds during the growth phase would be considerably different from that during the PHA accumulation phase. Therefore, under autotrophic conditions, determining the optimal concentrations and suitable supply strategies for NH_4_^+^ and PO_4_^3-^ is challenging but important for achieving efficient cell growth and PHA accumulation. In this study, alternating the addition of 3 M KOH and 14 *v/v*% NH_4_OH as the pH neutralizer was applied for this purpose.

The cell growth and PHA synthesis of *C. necator* under autotrophic conditions depend on the mass transfer of the substrate gases in the culture medium, which are affected by the rates of bubbling flow and stirring within the fermenter. In the cultivation of *C. necator* MF01/pBPP-ccr_Me_J_Ac_-emd as well as other aerobic bacteria, a high agitation speed and high aeration rate enhance the foaming of the culture broth, which accelerates the risk of outflow from the fermenter, particularly at high cell concentrations. Therefore, an antifoaming agent was added to prevent the outflow of the culture broth. However, antifoaming agents are surfactants and thus often show inhibitory effects on bacterial cells and exert insolubilizing effects on the gases in the culture medium. In this study, a slight amount of the antifoaming agent solution was repeatedly added into the medium whenever foaming was likely to cause an outflow. Unfortunately, the outflow of the culture broth was not completely repressed in the last cultivation cycle in this study, which achieved the highest cell concentration (Figure 5 and Figure 6) and resulted in a loss of 20 mL culture broth. As we observed that the required amount of the antifoaming agent varied for each cultivation even under the same conditions, the results of very-high-cell-density cultivation of MF01/pBPP-ccr_Me_J_Ac_-emd were rather difficult to reproduce. In order to achieve highly efficient production of PHBHHx from CO_2_ and H_2_, it will be necessary to optimize the concentrations of N- and P-sources in the medium and establish suitable feeding strategies during the cultivation.

The concentrations of the cells and PHBHHx obtained in this study are the highest among those previously reported for the autotrophic production of PHAs by employing hydrogen-oxidizing bacteria. Moreover, the significance of this study is that this cultivation system enables efficient production of the practical copolyester PHBHHX from CO_2_. However, the fermentation time was still longer than that of P(3HB) production. For example, Volova et al. reported that, in the autotrophic cultivation of *C. necator* B-10646 using a gas mixture composed of H_2_/O_2_/CO_2_ = 70:20:10 *v/v*%, they obtained a DCW of 50 g·L^−1^ and P(3HB) of 42.5 g·L^−1^ after 70 h [51]. We previously achieved a DCW of 91.3 g·L^−1^ and P(3HB) of 61.9 g·L^−1^ after 40 h cultivation of *C. necator* ATCC 17697^T^ by gas fermentation with high *k*_L_*a* of 2,970 h^−1^ while the O_2_ concentration was maintained below 6.9 *v/v*% [30]. In heterotrophic jar cultivation of recombinant *C. necator* constructed by Kaneka researchers, the DCW and PHBHHx reached approximately 215 g·L^−1^ and 175 g·L^−1^, respectively, after 68 h by using palm kernel oil as the carbon source [50]. Further progress is required in the modification of the recombinant strain and culture technology to increase the efficiency of copolymer production from CO_2_ to a rate comparable to using vegetable oils.

The production rate of the cells of hydrogen-oxidizing bacteria and PHA under autotrophic conditions depends on the mass transfer of the substrate gases in the culture medium. The oxygen transfer rate is particularly important because the DO limitation is inevitable at high cell concentrations [26]. We determined the stoichiometry during the exponential growth phase and the production phase of the homopolymer P(3HB) of a *C. necator* wild strain. The bacterium consumes the gases according to the following formula [18,19].

For exponential cell growth:

21.36H_2_ + 6.21O_2_ + 4.09CO_2_ + 0.76NH_3_ → C_4.09_H_7.13_O_1.89_N_0.76_ + 18.7H_2_O (1)

For P(3HB) production:

33H_2_ + 12O_2_ + 4CO_2_ → C_4_H_6_O_2_ + 30H_2_O (2)

The *k*_L_*a* value of the 1 L-scale fermenter with the basket-shaped agitation unit used in this study was about two times higher than that of the jar fermenter with the two-blade-type magnetic stirrer bar and rotating shaft used in our previous study [26]. However, the *k*_L_*a* of the larger agitation unit used in the 2 L-scale fermenter, which enabled us to increase the concentrations of the cells and P(3HB) up to 91.3 g·L^−1^ and 61.9 g·L^−1^, respectively, after 40 h under autotrophic cultivations, was remarkably high, reaching 2970 h^−1^ [30]. On the other hand, the *k*_L_*a* of the fermenter used in this study (450 h^−1^) was not very high for autotrophic culture of hydrogen-oxidizing bacteria; nevertheless, a very high cell yield was obtained over a relatively short cultivation period. This value was measured in the mineral medium without the cells. During the cultivation, fine bubbles innumerably increased in the culture medium as the cell concentration increased, and therefore, the mass transfer was enhanced at high cell concentrations. However, the mass transfer coefficient was not measured during the cultivation. Taking into consideration the *k*_L_*a* of 450 h^−1^ for the present fermenter, the production of cells and PHBHHx from CO_2_ is expected to be improved by using a reactor with a higher *k*_L_*a*, although it will be necessary to consider countermeasures against foaming and the resulting outflow of the culture broth.

In the RGCC system used in this study, safety measures like explosion proof-type compressors and electric devices, as well as oil-free treatment within the tubes for circulating the gas mixture, were not implemented, although they were used in the bench plant system for the production of the high-concentration homopolymer P(3HB) by employing a wild strain (type strain) of *C. necator* [30]. Preventing the detonation of hydrogen is quite an important issue for large-scale PHBHHx production from CO_2_. One of the strategies used aims to keep the O_2_ concentration of the gas mixture below 4 *v/v*%, which is lower than explosive limit, within the whole of the cultivation system. However, this conflicts with efficient production because such a low O_2_ concentration drastically reduces the oxygen transfer rate. There have already been several studies on the production of PHA from CO_2_ at an O_2_ or H_2_ concentration lower than the explosive limit [40,41,42,43,45,55], including a case using pressurized reactors [44]. The development of safe gas fermentation systems with high productivity represents a great challenge, and we are now investigating an explosion proof-type cultivation system incorporating these safety measures to safely produce PHBHHx from CO_2_ by maintaining the O_2_ concentration in the gas phase below the lower limit for detonation.

Additionally, in autotrophic cultivation of hydrogen-oxidizing bacteria, attention should be paid to the water produced by hydrogen oxidation. In our cultivation of MF01/pBPP-ccr_Me_J_Ac_-emd, by which the highest cell concentration was obtained (shown in Figure 5c), the volume of the water produced within the fermenter was estimated to be about 250 mL. Water dilutes the culture broth in the fermenter, which suppresses the increase in the concentrations of the cells and the polymer. The highest cell concentration (DCW, 109.5 ± 0.30 g·L^−1^) achieved in this study may be close to the “upper limit” that can be obtained in the autotrophic cultivation of hydrogen-oxidizing bacteria. Metabolic modifications of the hydrogen-oxidizing bacteria to increase the yields of cells and PHBHHx per hydrogen consumption will contribute to reducing water production as well as the cost for polymer production.

## Figures and Tables

**Table 1:** The bacterial strains and plasmids used in this study.

Strain	Relevant Marker	References or Resources
*C. necator* H16	Wild type	DSM 428
*C. necator* MF01	H16 derivative; *ΔphaC*::*phaC_NSDG_*, *ΔphaA*::*bktB*	[48]
Plasmid		
pBPP	pBBR1MCS-2 derivative; *P_phaP1_*, *T_rrnB_*	[48]
pBPP-ccr_Me_J_Ac_-emd	pBPP derivative; *ccr_Me_*, *phaJ_Ac_*, *emd_Mm_*	[13]

*phaA*, the short chain length (*scl)*-specific β-ketothiolase gene; *bktB*, broad-substrate-range β-ketothiolase gene (medium-chain-length (*mcl*)-specific β-ketothiolase); *phaC*, PHA synthase gene in the *pha* operon on chromosome 1; *phaC_NSDG_*, a gene of the N149S/D171G double mutant of broad-substrate-range (from C_4_ to C_7_) PHA synthase from *Aeromonas cavaie*; *P_phaP1_*, promoter region of *phaP1*; *T_rrnB_*, transcription terminator region from *E. coli*; *ccr_Me_*, crotonyl-CoA carboxylase/reductase gene from *Methylorubrum extorquens*; *emd_Mm_*, a codon-optimized gene encoding ethylmalonyl-CoA decarboxylase from *Mus musculus*; *phaJ_Ac_*, *scl*-specific (*R*)-2-enoyl-CoA hydratase gene from *A. caviae.*

**Table 2:** Cell growth and PHBHHx accumulation by the jar cultivation of *C. necator* MF01/pBPP-ccr_Me_J_Ac_-emd under the chemoautotrophic condition with different compositions of the mineral medium.

Initial Concentration in Mineral Medium		Cultivation Time (h)	Cell Concentration (OD_600_)	PHBHHx Content in Cells (*w/w*%)	Residual Concentration	
(NH_4_)_2_SO_4_ (g·L^−1^)	KH_2_PO_4_ (g·L^−1^)				PO_4_^3−^ (mg·L^−1^)	NH_4_^+^ (mg·L^−1^)
0.5	0.3	94	11.1	82.2 ± 1.8	273.0 ± 7.2	26.0 ± 1.2
1.0	0.5	52	68.0	85.5 ± 2.1	14.0 ± 3.5	1.2 ± 0.0
2.0	2.5	101	76.0	78.1 ± 1.9	1560.0 ± 29.0	14.0 ± 0.1
3.0	0.5	77	102.2	83.9 ± 0.9	67.0 ± 2.1	0.5 ± 0.0
3.0	1.0	115	96.0	82.2 ± 2.6	242.0 ± 3.9	68.0 ± 1.9
5.0	0.5	78	117.1	80.5 ± 2.9	28.2 ± 0.6	2.0 ± 0.0
10.0	1.0	91	94.5	39.5 ± 3.1	1.0 ± 0.1	61.0 ± 2.1
10.0	4.0	72	70.3	48.8 ± 0.7	1720.0 ± 36.0	30.1 ± 0.6

## Data Availability

Data are contained within the article.
